# Predicting the diabetic foot in the population of type 2 diabetes mellitus from tongue images and clinical information using multi-modal deep learning

**DOI:** 10.3389/fphys.2024.1473659

**Published:** 2024-12-03

**Authors:** Zhikui Tian, Dongjun Wang, Xuan Sun, Chuan Cui, Hongwu Wang

**Affiliations:** ^1^ School of Rehabilitation Medicine, Qilu Medical University, Zibo, Shandong, China; ^2^ College of Traditional Chinese Medicine, North China University of Science and Technology, Tangshan, China; ^3^ College of Traditional Chinese Medicine, Binzhou Medical University, Yantai, Shandong, China; ^4^ School of Clinical Medicine, Qilu Medical University, Zibo, Shandong, China; ^5^ School of Health Sciences and Engineering, Tianjin University of Traditional Chinese Medicine, Tianjin, China

**Keywords:** diabetic foot, tongue features, objectified parameters, prediction model, machine learning

## Abstract

**Aims:**

Based on the quantitative and qualitative fusion data of traditional Chinese medicine (TCM) and Western medicine, a diabetic foot (DF) prediction model was established through combining the objectified parameters of TCM and Western medicine.

**Methods:**

The ResNet-50 deep neural network (DNN) was used to extract depth features of tongue demonstration, and then a fully connected layer (FCL) was used for feature extraction to obtain aggregate features. Finally, a non-invasive DF prediction model based on tongue features was realized.

**Results:**

Among the 391 patients included, there were 267 DF patients, with their BMI (25.2 vs. 24.2) and waist-to-hip ratio (0.953 vs. 0.941) higher than those of type 2 diabetes mellitus (T2DM) group. The diabetes (15 years vs. 8 years) and hypertension durations (10 years vs. 7.5 years) in DF patients were significantly higher than those in T2DM group. Moreover, the plantar hardness in DF patients was higher than that in T2DM patients. The accuracy and sensitivity of the multi-mode DF prediction model reached 0.95 and 0.9286, respectively.

**Conclusion:**

We established a DF prediction model based on clinical features and objectified tongue color, which showed the unique advantages and important role of objectified tongue demonstration in the DF risk prediction, thus further proving the scientific nature of TCM tongue diagnosis. Based on the qualitative and quantitative fusion data, we combined tongue images with DF indicators to establish a multi-mode DF prediction model, in which tongue demonstration and objectified foot data can correct the subjectivity of prior knowledge. The successful establishment of the feature fusion diagnosis model can demonstrate the clinical practical value of objectified tongue demonstration. According to the results, the model had better performance to distinguish between T2DM and DF, and by comparing the performance of the model with and without tongue images, it was found that the model with tongue images performed better.

## Introduction

We have witnessed during the past few decades, the chronic metabolic disorder, Diabetes Mellitus (DM) becoming a global health hazard (Blaslov et al., 2018). DM is a chronic condition in which high levels of blood sugar are caused by impaired insulin secretion or insulin resistance, or a combination of both (Lavery et al., 2007). Besides impacting the quality of life, DM has been known to lead to numerous complications with the expectancy of the life of the afflicted (Pedras et al., 2016; Saluja et al., 2020). Some complications associated with diabetes, including stroke, blindness, heart attack, lower limb amputation, and kidney failure, can increase mortality while decreasing quality of life (Cole and Florez, 2020; Bordianu et al., 2018). Nonetheless, one of the major complications of DM are the Diabetic Foot Ulcers (DFU), afflicting almost one-third of patients with diabetes in their life (Armstrong et al., 2017; Lim et al., 2017). DFU being more prevalent in women as compared to men, in type 2 DM, its global prevalence stands at 6.3% as per reports (Zhang et al., 2017). Moreover, the DFU also has a high rate of recurrence, with the values scaling 40% and 65% within a year and three, respectively (Armstrong et al., 2017). Hence, strategies for the prevention of DFU need to be established by the studies, imperatively (Armstrong et al., 2017; Lim et al., 2017). Nevertheless, with lesser adverse reactions and conspicuous therapeutic impacts, the TCM has been receiving immense attention. Recently, the demand for human-computer integrated health detectors has been gaining ground with substantial progress being made in the research of the treatment of DF by TCM. One of the characteristic methods of diagnosis by TCM is the diagnosis of the tongue, providing the visual detection of the patients. However, numerous factors impact the diagnosis leading to the insufficient objectification of the results. Nonetheless, the needs of effective tongue diagnosis by TCM could be achieved to a certain extent with the successful development of a series of models of the tongue images by the several scholars exploring the objectivity of the TCM diagnosis of the tongue since the advent of the 20th century. Becoming a research hotspot gradually, the applications of the instruments of the tongue have been attracting the attention of the clinical staff. For example, the ‘WZX system of analysing the colour of the tongue’ developed through several publications and diagnosis of the tongue by the Xiamen University and Shanghai University of Traditional Chinese Medicine (Li et al., 2004; Li et al., 2005). In medical diagnosis, the images of the tongue have been promoted by numerous research (Wang et al., 2005; Wang et al., 2004; Pang et al., 2005a; Pang et al., 2005b; Pang et al., 2004; Li and Yuen, 2002; Pham and Cai, 2004). However, the research on the intelligent system of diagnosis through TCM has been a late starter, and started as late as the mid-70s. In the learning of the algorithms of the machines, Deep Learning is the latest method, enabling the simulation of the human thinking by the machines. The TCM intelligent machines could be trained effectively for continuous progress and learning through deep learning, ensuring their effective and efficient practice in the diagnosis and treatment through TCM. To examine the understandability of the images by the machines, several tasks have been formulated over the years. The visual question answering has been one of the most widely watched tasks, which required the machine to provide correct answers to the image questions posed to it. Ease of evaluation and documentation from the cylinder sheet, was made possible through the visual question and answer system. Theoretically, considered as an AI-generated issue, the visual question-answering system could be applied as an alternative to the visual Turing test, with highly promising results (Zhuang et al., 2022). According to previous studies, it is particularly important in the case of diabetic foot complications that may increase the risk of overload damage due to alterations in tissue biomechanics (Naemi et al., 2017; Piaggesi et al., 1999; Chatzistergos and Chockalingam, 2022; Erdemir et al., 2006; Hsu et al., 2009). The ability to quantify the hardness of the plantar soft tissues can improve the prediction of diabetic foot ulcers and improve the clinical management of the disease (Naemi et al., 2017; Monteiro et al., 2023; Cw et al., 2020), so this study also included plantar stiffness as an indicator in this model for the first time.

This paper is based on the deep learning fusion model, designing a TCM tongue image recognition AI diabetic foot detector combining neural network and tongue image and answer technology, realize diabetic foot diagnosis in people with type 2 diabetes.

## Research content and methods

### Research objects

From 15 May 2019 to 15 October 2022, we collected 410 participants aged 18 and above from inpatients and outpatients of the TCM surgery and endocrinology departments of The Second Affiliated Hospital of Tianjin University of Traditional Chinese Medicine. By excluding subjects with incomplete information, there were 391 patients remaining, including 124 in the T2DM group and 267 in the DF group. We downloaded the electronic medical records of patients between 15 May 2019 and 15 October 2020, which allowed us to obtain information that could identify individual participants during the data collection period. All subjects were collected with sociological feature data, objectified tongue demonstration data and laboratory data. This study was approved by the Medical Ethics Committee of Tianjin University of Traditional Chinese Medicine (approval number: TJUTCM-EC20190004), and all participants had to provide written informed consent before the study’s initiation (S1).

### Diagnostic criteria

In this paper, according to the criteria stated in the diagnosis and treatment guidelines by the American Diabetes Association (ADA), the subjects were diagnosed with T2DM (American Diabetes Association Professional Practice Committee, 2022), fasting plasma glucose (FPG) ≥7.0 mmol/L and/or 2 h postprandial blood glucose ≥11.1 mmol/L, or were previously diagnosed as T2DM patients at The Second Affiliated Hospital of Tianjin University of Traditional Chinese Medicine.

The criteria for diagnosing DF refer to the Guidelines for DF Diagnosis and Treatment 2020 by the DF Expert Committee of the Chinese Chapter of the International Union of Angiology (Gu, 2020).

① Patients with a clear history of diabetes mellitus.
② Neuropathy: The skin of the affected limb is dry and sweat free, with tingling, numbness, burning pain, reduced or absent sensation at the extremities. The extremities show a stocking-like change, and there is a feeling of cotton wool on the feet while walking. 10g nylon filament test or 128HZ tuning-fork positive combined with abnormal ankle reflex, pain sensation and temperature sensation are used to assist in the examination of lower limb neuropathy.
③ Lower limb ischemia: Dry skin, poor elasticity, malnutrition, skin pigmentation, low temperature, and decreased or absent extremital arterial pulsation may be accompanied by intermittent claudication symptoms and resting pain in lower limbs, ulcers in the heel or metatarsophalangeal region, gangrene in the toe region, limb infections in some patients. Visual examination is used to observe skin abnormalities and foot deformities. Palpation is used to determine decreased or absent dorsal foot artery pulsation. Moreover, vascular murmurs in arterial stenosis are detected by auscultation. The above methods are used for the manifestation of lower limb ischemia.

If patients have any of ①, ② and ③, they can be diagnosed with DF.

### Case inclusion criteria

(1) Patients who meet the above diagnostic criteria; (2) The age range between 18 and 85; (3) Patients willing to cooperate in completing questionnaire survey and tongue image collection Cooperate to complete the questionnaire survey and tongue imaging collection; (4) Patients who sign informed consent form.

### Case exclusion criteria

(1) Patients with type 1 diabetes mellitus or acute complications of diabetes mellitus; (2) Patients who suffer from tumors, hematological system disorders and other serious diseases; (3) Patients severely affected by diet, drugs or diseases; (4) Patients who are unable to obtain complete information.

### Research content

Sociological indicators: age, gender, cigarette smoking history, diabetes duration, alcohol consumption history, and hypertension duration.

Physical indicators: BMI takes the measurement value of height and weight and is calculated by the equation (Eq.) BMI = weight (Kg)/height (m) (Lavery et al., 2007). The waist-to-hip ratio (WHR) is obtained by the equation WHR = waist circumference (cm)/hip circumference (cm).

Objectified tongue features: TC-a1, TC-R2, TC-G2, TC-L2, TC-a2, TC-L3, TC-R4, TC-B4, TC-b4.

Plantar hardness indicators: collecting 6 positions on the same side of the foot, with the unit of (HA).

Laboratory indicators collected via electronic medical records:

Blood glucose indicators: fasting plasma glucose (FPG), two hours postprandial blood glucose (P2BG), glycosylated hemoglobin (HbA1c)
Renal function indicators: creatinine (Cr), uric acid (UA)
Liver function indicators: alanine aminotransferase (ALT), aspartate aminotransferase (AST), alkaline phosphatase (ALP), γ-glutamyl transpeptidase (γ-GT)
Blood lipid indicators: Total cholesterol (TC), triglycerides (TG), Low density lipoprotein cholesterol (LDL-C), high density lipoprotein cholesterol (HDL-C)
Blood routine indicators: White blood count (WBC), red blood count (RBC), hemoglobin (HGB), Neutrophil count (NEUT)
Urine routine indicators: protein (PRO), glucose (GLU), ketone (KET), white blood cell (WBC)

### Research methods

#### Statistical analysis

Data will be analyzed for statistically significant differences by first checking that there is a Gaussian distribution and normally distributed continuous data were presented as mean (±standard deviations), and differences between groups were assessed by independent samples t-test. If the non-conforming normal distribution is expressed by the quartiles M(P25, P75), minimum (min), and maximum (max), the Chi-square test and Wilcoxon rank-sum test was used for comparison between groups. All tests were two-sided, with P < 0.05 indicating that the difference was statistically significant.

#### Investigator collection

First of all, professional training was conducted for researchers. Two researchers collected patient information according to the Basic Information Sheet and the TCM Clinical Diagnosis Record Form (S2) prepared by the team of the national key research and development plan.

#### Tongue manifestation review

Based on the *Diagnostics of Chinese Medicine*, two experienced TCM experts were asked to review the tongue manifestations simultaneously. In the event of a disagreement, a third expert would decide the outcome (wu, 2006).

#### Parameter setting of tongue diagnostic instrument

**FIGURE 1:**
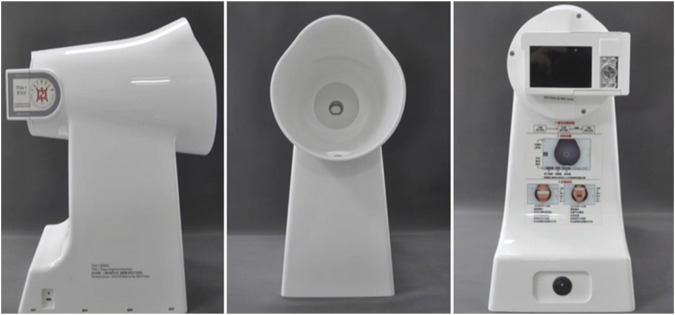
Appearance of TFDA-1 tongue diagnostic instrument.

We used TFDA-1 digital tongue diagnosis instrument developed by Intelligent Diagnostic Technology Research Laboratory of Shanghai University of TCM to collect tongue features data of participants. Main parameter settings: The light source was a cold white LED lamp (KA-3 021HVR4D1Z1S-C1-SH), with a color temperature of 0–6466K and an illumination of −2354lux, the CCD device was Eolane A12, and the lighting conditions were kept constant. The TFDA-1 tongue diagnostic instrument is shown in Figure 1.

#### Collection requirements for tongue images

Tongue images were taken in the morning when no food was eaten. A photographer was slightly higher than the subjects when shooting. The subjects could sit upright or lie on their back, facing a natural light source. A researcher first set the shooting parameters and disinfected the area where the tongue diagnostic instrument would contact their faces and jaws with alcohol. The collected subjects were instructed to maintain emotional stability, put their jaws against the corresponding position on the tongue diagnostic instrument, sit upright, lean slightly forward, easily extend their tongues to relax, expand their lingual surfaces, and press their tongue tips down slightly. Then, the researcher clicked the position of tongue middle on the instrument screen to complete the collection.

#### Tongue image segmentation and parameter interpretation

**FIGURE 2:**
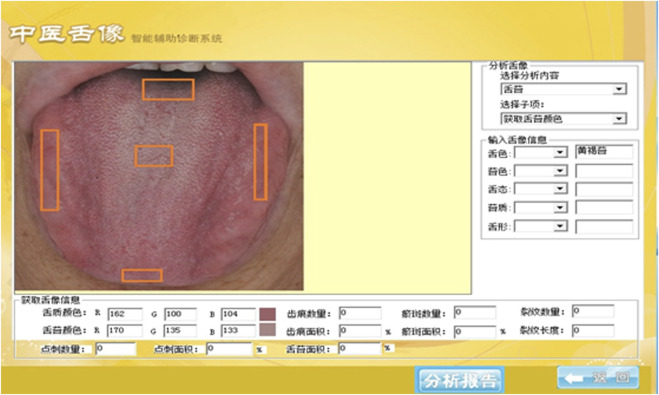
Tongue image intelligent auxiliary diagnosis system of traditional Chinese medicine.

**FIGURE 3:**
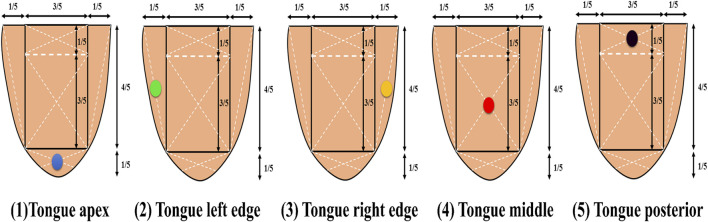
Five measuring points of the tongue.

The tongue image segmentation and parameter acquisition were obtained through the tongue image intelligent auxiliary diagnosis system of traditional Chinese medicine, which took several shots, deleted the problematic images, and only kept the perfect ones. Tongue color indicators were derived from the two spaces, RGB and CIE L*a*b*, and the RGB indicators corresponding to the tongue could be obtained through the intelligent auxiliary diagnosis system (Figure 2). The values of R (Red), G (Green), and B (Blue) ranged from 0 to 255, where “0” indicated that the color component was not present, and “255” indicated that the color component was saturated ^[63]^. Color was converted from RGB to L*a*b*, where L* represented brightness with its value ranging from 0 to 100 (black to white axis), and a* and b* represented chromaticity, with a* value ranging from 127 to −128 (red to green axis) and b* value from 127 to −128 (yellow to blue axis) (Duan et al., 2018; Su et al., 2011; Cade, 2008). Tongue color (TC) at five measuring points of the tongue was collected for research (Figure 3). According to common clinical practices and TCM diagnostics, the RGB indicators were screened: tongue middle (TC-R4, TC-B4), tongue left edge (TC-R2, TC-G2), and the L*a*b* indicators: tongue apex (TC-a1), tongue left edge (TC-L2, TC-a2), tongue right edge (TC-L3), tongue middle (TC-b4). The color change can be calculated in the following equations (Equations 1–3):

$$\left[\begin{array}{l} X\\ Y\\ Z \end{array}\right]=\left[\begin{array}{ccc} 0.412453 & 0.357580 & 0.180423\\ 0.212671 & 0.715160 & 0.072169\\ 0.019334 & 0.119193 & 0.950227 \end{array}\right]\left[\begin{array}{l} R\\ G\\ B \end{array}\right]$$ (1)

Then, the conversion from the CIEXYZ color space to the CIE L*a*b* color space is completed in the following equations:

$$\begin{array}{l} L^{*}=116f\left(Y/Y_{n}\right)-16\\ a^{*}=500\left[f\left(X/X_{n}\right)-f\left(Y/Y_{n}\right)\right]\\ b^{*}=200\left[f\left(Y/Y_{n}\right)-f\left(Z/Z_{n}\right)\right] \end{array}$$ (2)

$$f\left(t\right)=\left\{\begin{array}{ll} t^{1/3} & \text{ if }t>{\left(\frac{6}{29}\right)}^{3}\\ \frac{1}{3}{\left(\frac{29}{6}\right)}^{2}t+\frac{4}{29} & \text{ otherwise } \end{array}\right.$$ (3)

#### Plantar hardness data collection

**FIGURE 4:**
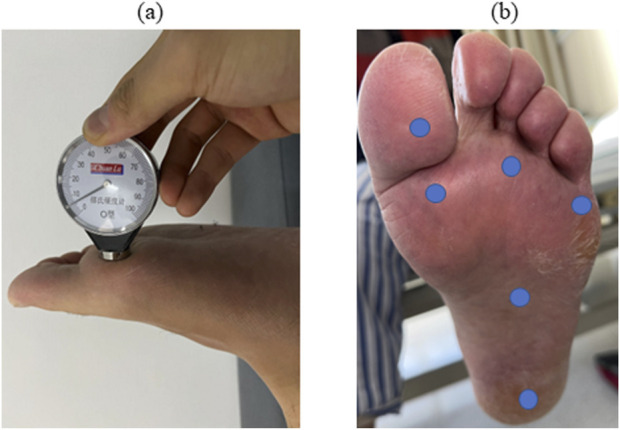
Plantar hardness collection. Note: **(A)** shows the usage of a Shore hardness tester. **(B)** Shows the 6 measuring points of the foot, from top to bottom, from left to right, including the big toe pulp, the first metatarsophalangeal joint, the third metatarsophalangeal joint, the fifth plantar metatarsophalangeal joint, the plantar middle, and the heel.

All data was collected in the afternoon. The right foot data was collected in T2DM patients, while the left side was collected in DF patients with ulcers on the right foot. Patients were required to keep their feet out of contact with water-like liquids for 2 h, take off their socks, and rest in bed for 5 min. Then, a Shore hardness tester (Shanghai Luchuan, O-type, measuring range 10-90HA) was used for measurement. For the same patient, we measured 6 points successively, the big toe pulp → the first metatarsophalangeal joint → the third metatarsophalangeal joint → the fifth metatarsophalangeal joint → the plantar middle → the heel (Figure 4). Moreover, the mean values were obtained by repeating the measurement twice. Laboratory indicators were collected via electronic medical records.

#### Sample size estimate

The sample size is estimated based on a rough sample size formula (Riley et al., 2020).10 events per variable (EPV) is widely used in the estimation of clinical prediction models, and the preliminary estimation of the independent variables was 39 items, and 10 patients were needed for each independent variable. Therefore, the sample size required for this study is 39 × 10 = 360 cases. In this study, 267 DF patients and 127 T2DM patients from The Second Affiliated Hospital of Tianjin University of Traditional Chinese Medicine who met the inclusion and exclusion criteria from April 2019 to October 2022 were selected for the study.

#### Missing value handling

We confirmed the method of filling the missing values according to the data type, and performed interval estimation and rough estimation. Mode interpolation was used to interpolate the missing values in such content as tongue features, marriage, and education level. Mean value interpolation was used to interpolate the missing values of continuous variables, such as FPG and P2BG.

#### Evaluation indicators

Four indicators, namely Accuracy, Sensitivity, Specificity, and F1-score, were mainly used in this paper. Accuracy, as the most common indicator, represents the ratio of correct sample size to total sample size in the model. The higher the value is, the better the classifier performance will be. Sensitivity indicates the proportion of accurately diagnosed DF patients in all DF patients. The higher the value is, the stronger the classifier’s ability to identify positive samples will be. Specificity refers to the model accuracy in predicting T2DM patients among all results of T2DM patients, and is considered as a measure of the classifier’s ability to recognize T2DM patients. F1-score, also known as balanced F Score, is the comprehensive result of Precision and Recall. True Positive (TP) means that a person is accurately interpreted as a DF patient. True Negative (TN) means that a person is accurately interpreted as a T2DM patient. False Positive (FP) means that a T2DM patient is misinterpreted as a DF patient. False Negative (FN) means that a DF patient is misinterpreted as a T2DM patient. Accuracy, Sensitivity, Specificity, and F1-score can be calculated in Equations 4–7, respectively.

$$Accuracy=\frac{TP+TN}{TP+TN+FP+FN}$$ (4)

$$\text{Sensitivity}=\overset{\text{TP}}{\underset{\text{TP}+\text{FN}}{—}}\times 100\%$$ (5)

$$\text{Specificity}=\overset{\text{TN}}{\underset{\text{TP}+\text{FP}}{—}}\times 100\%$$ (6)

$$F-1=\frac{2\times Precision\times Recall}{Precision+Recall}$$ (7)

The deep learning architecture essentially refers to a classifier. In addition, Confusion Matrix is a commonly used method to evaluate the classification level in the dichotomous problem of identifying DF patients in diabetes population. True Positive (TP) refers to the number of samples labeled as DF among the DF patients. False Negative (FN) refers to the number of samples labeled as T2DM among the DF patients. False Positive (FP) refers to the number of patients labeled as DF among the actual T2DM patients. True Negative (TN) refers to the number of patients labeled as T2DM among the actual T2DM patients. Therefore, the confusion matrix can be considered as a table of two rows and two columns composed of TP, FN, FP, and TN. The confusion matrix refers to a table for summarizing the predictions of a classification model through summing up the records in the dataset according to true and predicted categories. The rows of the confusion matrix represent the true category of the samples, and the number of the samples in each row represents the true number for that category. The columns of the confusion matrix represent the predicted category of the samples, and the number of the samples in each column represents the predicted number for that category. The confusion matrix in binary classification is shown in Table 1.

**TABLE 1:** Confusion matrix.

		True value	
		Positive	Negative
Predicted Value	Positive	True Positive	False Positive
	Negative	False Negative	True Negative

#### Experimental environment

The Python 3.7 deep learning environment was used in this experiment, with the specific configuration shown in Table 2.

**TABLE 2:** Experimental environment configuration.

Framework version	Pytorch
GPU	16 GB
VGA Card	Tesla V100
Host Memory	32 GB
CPU	i7 10700K
OS	windows10

#### Experimental dataset

In the dataset, data was labeled into two categories: T2DM and DF, with the image size of 512 × 512 and the format of TIFF. The dataset was divided into training dataset and testing dataset. The training dataset contained 311 images for algorithm development. The testing dataset contained 80 images for the verification of algorithm.

#### Normalization processing

Normalization refers to the process of mapping data to a specified range, typically [0,1] and [−1,1]. It is aimed to remove the dimensions and dimensional units of data in different dimensions (Li, 2021). The common normalization function is Max-Min function. Let the data be x = [9,3,4,5, … … ]n, then the normalized x: x = (x-min(x))/(max(x)-min(x)).

#### Multi-mode deep learning prediction model

From the analysis of the TCM risk factors of DF in the first two parts, it can be seen that the DF diagnosis is characterized by multimodality. In addition to the influence of life style, tongue characteristics and tongue color indicators can assist in the DF clinical diagnosis. Doctors generally diagnose DF by combining multimodal pathological information (laboratory data and foot characteristic data, etc.), which can put forward high requirements for doctors’ clinical experience and level. According to the TCM diagnosis on DF, we constructed a multi-mode ResNet50 network structure integrating objectified TCM data, plantar hardness data and laboratory characteristics in this study.

In this study, we applied the design of a ResNet50 model trained on the ImageNet. ResNet50 refers to a convolutional neural network (CNN) model with unique structure and excellent performance, consisting of 50 convolutional layers and fully connected layers, with a parameter size of 25 million and a stack of 3 × 3 convolutional layers in its structure, but the idea of residual network is incorporated, so that gradients can be transmitted across layers, which is characterized by the fact that the two output convolutional layers can be circled to the next layer during input. By increasing the number of layers, the model can be more stable and powerful in transmitting information, and more advanced and abstract features can be obtained, thereby improving the effectiveness of the model, together with such advantages as high precision and easy optimization (Dong, 2021). In this study, we proposed a multi-mode DF model based on ResNet50.

**FIGURE 5:**
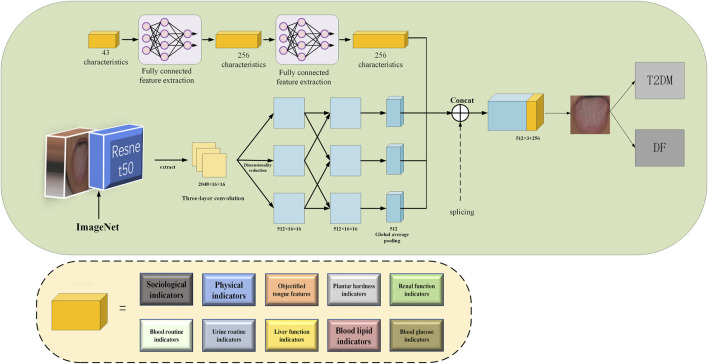
Model flowchart.

The constructed ResNet50 network structure is shown in Figure 5. Firstly, the tongue image dataset was collected. Then, tongue images were preprocessed. Three convolution layers of the last stage were removed after the image preprocessing, with a tongue image size of 2048 × 16 × 16. Furthermore, the features of different layers were extracted from the tongue images with a size of 2048 × 16 × 16, and the feature dimension was reduced at the same time. The image size of the upper layer was reduced to 512 × 16 × 16. The linear interaction between layers could improve the diversity of features. Images with a size of 512 were input into the average pooling layer. Meanwhile, a fully connected layer was used to incorporate baseline data, objectified tongue image data, laboratory data, and plantar hardness data into the model for feature extraction. Through further splicing with the tongue features after global average pooling (GAP), the aggregate features, with a size of 512 × 3 + 256, were obtained. Finally, the spliced features were input into the classifier for DF diagnosis (Goodfellow, 2016). The output size of the classifier was 2, namely the two categories of T2DM and DF, corresponding to the probability of the two categories. In order to verify the importance of tongue images, we also compared the performance results of the model that were not included in the tongue image with the results of this model. As a result, the category with the highest probability was the final predicted result. In order to fully verify the robustness of the model, we set up five different sets of random seeds on the dataset to verify the generalization ability of the model.

## Results

### Clinical features of T2DM an DF

A total of 391 people were included in this study, and they were classified into two groups according to whether they had DF or not, including 124 T2DM patients and 267 DF patients. Most of the DF patients were male, accounting for 65.2%. The age in the DF group was older than 67 (62, 72) in the T2DM group. In addition, the BMI and WHR in the DF group were higher than those in the T2DM group, and the durations of diabetes and hypertension were significantly higher than 15 (9, 20) and 10 (0, 20) in T2DM group. Laboratory results were not equal in both groups. It can be seen from Table 3 that the plantar hardness of DF patients was higher than that of T2DM patients in the 6 parts, including the big toe pulp, the first metatarsophalangeal joint, the third metatarsophalangeal joint, the fifth metatarsophalangeal joint, the plantar middle, and the heel. Among RGB parameters, the values of TC-R2, TC-G2, TC-R4 and TC-B4 in the T2DM group were higher than those in the DF group.

**TABLE 3:** Statistical analysis of two sets of parameters [Mean (SD), Median (P25, P75)].

Variables	T2DM n = 124	DF n = 267	*p*-Value
Age (years)	65 (59, 71)	67 (62, 72)	0.001
BMI	24.2 (23.5, 26.6)	25.2 (22.9, 27.4)	0.073
WHR	0.941 (0.891, 0.98)	0.953 (0.909, 0.98)	0.260
Diabetes Duration (years)	8 (2, 15.3)	15 (9, 20)	<0.001
Hypertension Duration (years)	7.5 (0, 14.3)	10 (0, 20)	0.658
Gender (n,%)			0.001
Male	58 (46.8)	174 (65.2)	
Female	66 (53.2)	93 (34.8)	
Cigarette Smoking History			0.031
No	90 (72.6)	164 (61.4)	
Yes	34 (27.4)	103 (38.6)	
Alcohol Consumption History			<0.001
No	110 (88.7)	168 (62.9)	
Yes	14 (11.3)	99 (37.1)	
TC-a1	17 (15, 18)	17 (16, 18)	0.503
TC-R2	158 (153, 167)	156 (145, 165)	0.026
TC-G2	91 (85, 100)	90 (81, 98)	0.151
TC-L2	70 (68, 73)	70 (68, 73)	0.643
TC-a2	16 (15, 18)	16 (14,18)	0.045
TC-L3	69 (67,72)	69 (67,72)	0.859
TC-R4	152 (136,163)	147 (123,161)	0.158
TC-B4	94 (79,105)	90 (79,106)	0.458
TC-b4	3 (2,4)	3 (2,4)	0.719
The Big Toe Pulp Hardness	10 (8,12)	13 (10,15.5)	<0.001
Hardness at the First Metatarsophalangeal Joint	12 (10,15)	15 (12,18)	<0.001
Hardness at the Third Metatarsophalangeal Joint	12 (10,15)	14 (12,18)	<0.001
Hardness at the Fifth Metatarsophalangeal Joint	13 (10,15)	15 (12,20)	<0.001
The Plantar Middle Hardness	12 (10,14.3)	15 (12,19)	<0.001
The Heel Hardness	19 (16,21.3)	21 (18,25.5)	0.003
FPG (mmol/L)	8 (7.1,10.3)	7.7 (7,9.71)	0.111
P2BG (mmol/L)	11.4 (10,15)	11 (9.7,13.7)	0.107
HbA1c (%)	7.3 (6.8,8.4)	7.5 (6.8,8.56)	0.168
ALT (U/L)	17 (12,22)	17 (11,20)	0.323
AST (U/L)	16 (12.8,19)	16 (12,28)	0.042
ALP(U/L)	72 (55,90)	74 (64,83)	0.347
GGTAE (U/L)	20.5 (16,30)	26 (15,28)	0.367
TC (mmol/L)	3.8 (3.18,4.49)	4.28 (3.43,4.67)	0.097
TG (mmol/L)	1.69 (0.95,2.14)	1.43 (1.08,1.79)	0.897
LDL-C (mmol/L)	2.5 (1.81,3.29)	2.4 (1.95,2.96)	0.342
HDLC (mmol/L)	0.91 (0.83,1.01)	1.02 (0.85,1.1)	0.056
Cr (umol/L)	91.6 (60.5,107)	96 (66.1,101)	0.458
UA (umol/L)	332 (264,378)	331 (288,373)	0.826
PRO (n,%)	22 (17.7)	94 (35.2)	<0.001
GLU (n,%)	33 (26.6)	153 (57.3)	<0.001
KET (n,%)	8 (6.5)	78 (29.2)	<0.001
WBC(n,%)	9 (7.3)	67 (25.1)	<0.001
WBC (10^9^/L)	6.7 (5.75, 7.32)	7.7 (6, 8.3)	0.001
RBC (10^12^/L)	4.4 (4.22, 4.77)	4.6 (3.78, 4.75)	0.450
HGB (g/L)	127 (114, 143)	124 (114, 139)	0.384
NEUT (10^9^/L)	4.8 (3.77, 5.49)	6.34 (4.5, 22)	<0.001

### Model performance

**FIGURE 6:**
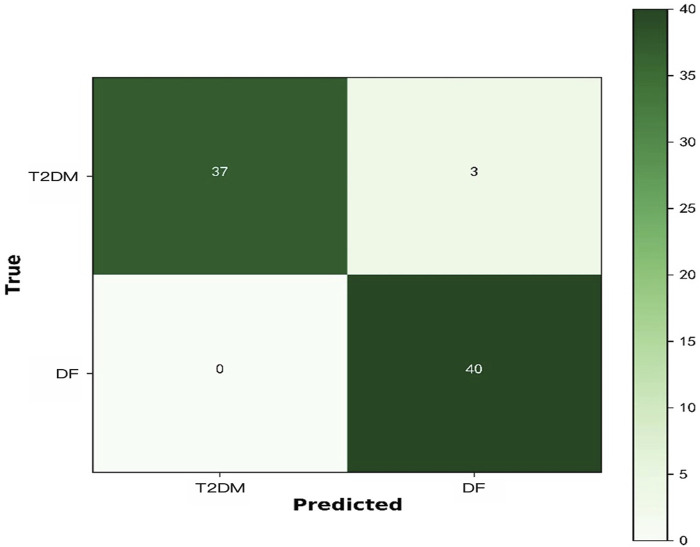
Confusion matrix.

As shown in Table 4 and Figure 6, ResNet50 performed well in classifying DF in this dataset, with Accuracy, Sensitivity, Specificity and F1-score all above 0.9. And a comparison was made with and without the tongue image. In order to fully verify the robustness of the model, we set up five different sets of random seeds on the dataset, and the results were 0.95, 0.975, 0.95, 0.9375, and 0.9375, respectively, indicating that the dataset has good generalization ability.

**TABLE 4:** Model performance results.

Evaluation indicators	With tongue image	Without tongue image
Accuracy	0.95 (0.8694–0.9993)	0.925 (0.5443–0.9804)
Sensitivity	0.9286	0.8696
Specificity	0.9737	1.0
F1-score	0.9392	0.8965

In this study, we applied the design of a ResNet50 network model trained on the ImageNet. In addition to meaningful sociological features and objectified tongue imaging data from previous studies, we also incorporated patients’ plantar hardness and tongue images into the model based on clinical significance, thereby significantly improving the algorithm performance. It can be proved that the multi-mode DF model based on the ResNet50 proposed in this study has been effective, because the convolutional network can extract image features, thus achieving a more accurate classification. The introduction of this technology can improve the performance of deep convolutional networks in the task of DF classification, which further proves that the optimization techniques adopted in this paper can help deep convolutional networks capture graphic features in a more effective way, so as to achieve better classification results of DF and diabetes complications.

Some studies have found that age and diabetes duration can exert an impact on vasculopathies in T2DM patients (Gottsäter et al., 2005). In elderly patients with a diabetes duration of more than 10 years, long-term exposure to hyperglycemia and insulin resistance or other risk factors, such as obesity, hypertension and hyperlipidemia, can accelerate the progression of atherosclerosis in diabetes patients, because hyperglycemia may lead to impairment of vascular endothelial cell function. With the prolongation of the T2DM duration, the risk of atherosclerosis in patients is also increasing. This is similar to the results of this study that the diabetes duration has been regarded as a risk factor for DF, with the duration of 10–20 years (OR 2.841, 95%CI 2.103–3.837); the duration of >20 years (OR 5.989, 95%CI 3.593–9.981) in the final model.

DF has been one of the important complications in elderly patients with diabetes. Elderly patients accounted for 67% of all DF amputees, and more than 30% of diabetic patients eventually developed into DF patients (Migdalis et al., 2017). There was a high risk of death after amputation, ranging from 40% within 3 years to 50%–70% within 5 years (Beyaz et al., 2017). According to the results of this study, the proportion of elderly patients with DF is significantly higher than that of patients with T2DM (32.8% vs. 17.4%), especially those over 70 years of age and with diabetes duration of more than 20 years who should be alert to the risk of DF disease. Additionally, a history of cigarette smoking has become a risk factor for DF (OR 2.187, 95%CI 1.618–2.956).

The smoke produced by cigarette combustion can enter the respiratory and digestive systems through breathing, and finally enter the blood circulation (Xia et al., 2019). With the continuous accumulation of chemicals in the body, they can eventually cause pathological damage to the skin (Álvaro-Afonso et al., 2018). Tracey et al. probed into the related factors of DF in the elderly, and found that smoking is one of the risk factors for ineffective wound treatment in diabetic foot ulcer (DFU) patients (Tracey et al., 2016). Another retrospective study explored the influencing factors of DFU recurrence, and found that smoking is a predictor of recurrent ulcers in DF patients (Huang et al., 2019). Meanwhile, DF amputations may also be related to smoking (Van et al., 2021). Smoking can cause vasoconstriction, hinder blood flow, and affect the healing of ulcer wounds (Xia et al., 2019).

## Discussion

Artificial intelligence (AI) refers to a system that realizes its goals by perceiving the environment and taking actions (Legg, 2007). Unlike the natural intelligence of humans or animals, it is a type of machine intelligence (Helm et al., 2020). AI, as a new science, is used to simulate and extend the methods, technologies, and theories of human intelligence, which coincides with the current demand for TCM modernization. With the rapid development of medical big data analysis and computer competency, AI has been widely used in various fields, such as medicine, due to its advantages of high efficiency, accuracy, and automation. Machine learning and deep learning have already become the two main categories of current AI approaches (Pannu, 2015). The former is identified by extracting features from raw data, and then it passes the information to a classifier for training to obtain a model. Deep learning, on the other hand, adjusts network parameters through the training of raw sample data, and then combines features to form high-level representations of attribute categories or features (Esteva et al., 2019). It not only eliminates the complicated and tedious process of machine learning, but also has stronger adaptability and convertibility, thus gaining advantages in task accuracy in multiple fields (LeCun et al., 2015), especially in visual recognition tasks, such as medical fields, including imaging and digital pathology (van Griethuysen et al., 2017; Niazi et al., 2019).

The concept of deep learning was put forward by Hinton et al., in 2006 (Hinton and Salakhutdinov, 2006), with multiple hidden layers and perceptron in its early architecture. However, due to the hardware limitations at that time, deep learning was in a relatively slow development. In recent years, with the upgrading of computer software and hardware, deep learning technology has been developed rapidly. As early as 2007, there were relevant studies on combining TCM tongue diagnosis with machine learning. With the popularity of deep learning technology, some scholars attempted to conduct tongue diagnosis research related to deep learning. Firstly, in terms of tongue segmentation, Li et al. utilized the CNN to segment the tongue based on the HSV color space (Li et al., 2017). A large number of studies have proved the feasibility of deep learning in the TCM tongue diagnosis research, as well as its high value of technology platform. However, tongue diagnosis is easily influenced by the external environment and the skills of doctors, so that many big data experiments are still needed to verify the clinical value of different deep learning models in the TCM tongue diagnosis.

Tongue diagnosis is considered as one of the important methods of the TCM inspection. As a non-invasive tool, it can provide an effective basis for clinical practice, so that doctors are required to make judgments in a short time based on visual observation and experience. Tongue demonstration refers to the tongue appearances observed by the naked eyes, which is mainly divided into three parts: tongue coating, tongue texture and sublingual veins. TCM believes that the human body is an organic whole. Through the rich clinical experience of medical professionals, it is found that the organs and tissues of the human body are interconnected with tongue demonstration and meridians, and that the human health and disease status can be dynamically reflected in the tongue appearances. As a result, the TCM diagnosis method of “inspecting the tongue for detecting diseases” has been formed for a long time. Tongue diagnosis can easily reflect a large amount of pathological information of the human body, and its results are easily limited by a doctor’s experience, diagnostic skills and knowledge reserves. Changes in external environmental conditions, such as temperature and light, can also exert an important impact on diagnostic results. Hence, the objective conditions and standard quantification in the process of tongue diagnosis have been very important. The establishment of objective indicators for tongue diagnosis can reflect the human functions or pathological conditions in a more accurate and objective manner, so that it is of great significance for the objectification and accurate interpretation of tongue diagnosis. In this study, the TFDA-1 tongue diagnostic instrument developed by the Intelligent Diagnostic Technology Research Laboratory of Shanghai University of Traditional Chinese Medicine was used to objectively collect the color values in five tongue points of DF patients (Shi et al., 2021; Li et al., 2021; Li et al., 2022a; Li et al., 2022b; Shi et al., 2022). The TFDA-1 is equipped with standard illuminants and high-definition (HD) cameras, which can maintain a constant light intensity and provide HD tongue images for post analysis.

At present, preliminary results have been achieved in tongue demonstration research. With the development of big data and TCM technology, there have been many studies based on big data to investigate the relationship between tongue color characteristics and diabetes mellitus or diabetes complications. Li et al. utilized the vector quantization variational auto encoder (VQ-VAE) to extract tongue image features of diabetes patients, and then used K-means to divide diabetes patients into four clusters, among which the highest classification accuracy was 87.8% (Li et al., 2022b). Yang et al. used the machine learning algorithm of random forest (RF) to classify T1DM and T2DM, with the classification accuracy of 0.85 (Xiang et al., 2021). Xia et al. collected the physicochemical and TCM indicators from 450 patients with metabolic syndrome, and selected the best performing model by comparing the performance of four machine learning models, among which the RF obtained the best result with an accuracy of 0.942 (Xia et al., 2021). These studies were aimed to classify and diagnose diseases. As far as we know, there is no research on the objectification of tongue color in DF patients, together with no evidence of a connection between L*a*b* and RGB values and DF.

Tongue color has been used as a qualitative indicator for the TCM diagnosis (Lo et al., 2015; Han et al., 2016; Pang et al., 2020; Zc et al., 2020). Some scholars have suggested that it is logical to take three coordinates, because color measurement is basically composed of a single point in space (Lee, 2000). However, in disease conditions, such as in clinical studies of tongue demonstration in diabetic patients, it is also feasible to use only one value (CIE a*) or two values (CIE a* and CIE b*) instead of three values. The feasibility of CIE L*a*b* tongue color in tongue demonstration classification has been demonstrated through comparing the CIE L*a*b* values in the existing literature (Yu et al., 2017). Duan et al. also found that there were differences in the b values of the L*a*b* color space between esophageal cancer patients and normal subjects (Duan et al., 2018). Similar studies have shown differences in laboratory values of tongue and tongue coating in lung cancer patients (Su et al., 2011). Among the diabetes-related studies, according to a study on oral microorganism, lower CIE a* values and higher CIE b* values in patients with metabolically associated fatty liver disease (MAFLD) were associated with carbohydrate metabolism disturbances and oral microbiome inflammation, resulting from the risk of microvascular or macrovascular complications caused by the prolonged diabetes duration and a high blood glucose level. In addition, most of the diabetes patients were found to have oral abnormalities, such as paradontosis, lost teeth, xerostomia, dental caries, burning mouth syndrome, dysfunctions of taste and salivary glands, delayed wound healing, oral lichen planus (OLP), geographic tongue, and candidiasis. A potential explanation for the connection between diabetes mellitus and tongue demonstration may be that the prolonged diabetes duration and a high blood glucose level can lead to microvascular or macrovascular complications (Cade, 2008). Moreover, most of the diabetes patients have different oral clinical manifestations, which can exert different impacts on tongue color (Ma et al., 2018). Hsu et al. showed that the areas of yellow fur, thick fur, and purple tongue were significantly larger in T2DM patients (with 199 T2DM cases and 372 non-diabetic cases as controls) than in non-diabetic patients. Thick fur, yellow fur, and purple tongue are more common in T2DM patients, indicating that tongue color can be used as a simple, non-invasive tool for pre-diabetes diagnosis (Pc et al., 2019). Therefore, the CIE L*a*b* color parameters were determined to observe the effect of tongue color on T2DM and its complications. In this study, the DF patients mostly showed lower R value (dark red), lower G value (dark green) on the tongue left edge and tongue middle, as well as higher B value (dark blue) on the tongue middle. According to the diagnosis theory of TCM, a purple-red tongue indicates excessive heat in the viscera or yin-fluid loss after a long illness. A dark tongue is often a clinical manifestation of severity as the condition worsens. A bluish-purple tongue, as the main syndrome of vital energy and blood stasis, is mostly caused by the intense heat toxin, the loss of body fluid, and the stagnation of vital energy and blood, which may be related to the DF causes.

As diabetes progresses, patients tend to lose a sense of protection from pressure and pain in their feet due to peripheral neuropathy. As a result, they repeatedly overload ulcer healing without paying attention, resulting in poor healing of open wounds, susceptibility to infection, and possibly even amputation (Tonna et al., 2024). Handheld hardness testers to measure shore hardness (SH) have been widely used to assess soft tissue biomechanics (Thomas et al., 2003; Charanya et al., 2004; Kissin et al., 2006; Aghassi et al., 1995; Holowka et al., 2019). Hardness is a unit of reading for a Shore hardness tester measurement, usually denoted as “HA”. SH can give dimensionless values between 0 and 100, with higher SH values indicating high indentation resistance, but SH interpretation is challenging due to the complex internal structure of soft tissues. A recent study analysis showed that SH is very sensitive to skin thickness, quantifying the deformable ability of tissues (Chatzistergos et al., 2022). Therefore, differences in the measurement of SH may be due to differences in subcutaneous tissue stiffness, but it is unclear whether SH is able to detect changes in clinically relevant differences. A statistically significant association was observed between SH in the T2DM group and the DF group. The stiffness of the six parts of the plantar in DF patients was greater than that in T2DM patients (*p* < 0.05). The six specific plantar sites selected for this study are consistent with where diabetic foot ulcers may occur, which have previously been studied in the literature using SH (Thomas et al., 2003; Charanya et al., 2004; Holowka et al., 2019). This first suggests that SH is affected by skin thickness and skin stiffness (Chatzistergos et al., 2022), but also suggests that the risk of DF is also associated with an increase in skin thickness. In addition, this simple, non-invasive form of measuring skin thickness and tongue color is well suited as a screening supplement for diabetic complications in the clinic.

A total of 391 cases were included in this study, including 267 DF patients, of which the majority were male. The average age, BMI and WHR of DF patients were higher than those of T2DM patients, and their durations of diabetes and hypertension were significantly higher than those of T2DM patients. It is worth noting that the hardness of the six points on the foot of DF patients was greater than that of T2DM patients. In this study, the ResNet50 network model was applied to identify DF in T2DM patients. Through the collection of tongue images, plantar hardness data, laboratory data, objectified tongue data and sociological feature data, we obtained the following results: Accuracy = 0.95, Sensitivity = 0.9286, and F1-score = 0.9392, indicating that the ResNet50 model has a good performance for DF identification. In order to further verify the importance of tongue images in the model, we compared the results obtained with the above results by not including tongue images, but only plantar hardness data, laboratory data, objectified tongue data, and sociological feature data, and the results showed that the models with tongue images were superior to those without tongue images. In this study, it is concluded that the diagnostic accuracy of DF with multi-feature fusion is higher than that with single feature, suggesting that if tongue images are fused with DF-related feature indicators, we may achieve a higher accuracy. In order to make use of the discriminant components between these tongue images, a general multi-feature learning method was designed by considering the common components and discriminant components between different feature domains. Experiments comparing the use of a single task and a typical fusion method confirm the superiority of our fusion method in the tongue image and the text dataset. The results of this study suggest that the proposed pipeline considering tongue image and multiple feature learning strategies may have generalizability on the dataset and contribute to accurate non-invasive DF diagnosis, with commercial applications.

On account of the deficiency of tongue image representation in traditional machine learning, we proposed a DF diagnosis algorithm based on deep learning in this paper. First of all, on the basis of the objectified data and sociological feature data of DF tongue demonstration screened in previous parts, plantar hardness data and laboratory data were added as the fully connected layers for feature extraction. Then, the tongue images of the last layer after the GAP of neural network were spliced with the features of the fully connected layer. Finally, they were input into the classifier for DF classification. Experiments on the DF dataset can demonstrate the effectiveness of the proposed algorithm. This proved that the multi-modal DF model based on ResNet50 proposed in this study was effective, because the convolutional network can extract the features of the image, so as to achieve more accurate classification. The introduction of this technique can improve the performance of the deep convolutional network in the diabetes classification task, which further proves that the optimization technique adopted in this study can help the deep convolutional network to capture the graph features more effectively, so as to achieve better classification results of diabetes and diabetes complications.

## Conclusion

This method has achieved good results in the DF classification of diabetic foot, with its performance indicators above 0.9 through analysis, mainly because of the combination of multi-mode data, including tongue images, objectified data, foot data, and laboratory data. For the training of neural networks, the data can exert a very important impact on the training performance. The ResNet50 model proposed in this paper not only has a high detection accuracy, but also can maintain strong robustness for different input types (images and data). In summary, the model can have a better detection performance through combining all the factors. Based on this study, we will develop apps and mini programs that are more suitable for clinicians, and predict the probability of diabetes complications in patients with T2DM by inputting the factors screened out in our study and the images of the patient’s tongue taken. Because we had screened for these risk factors for patients with diabetic complications, doctors only need to select the factors that we have screened for clinical use, reducing the workload of clinicians.

## Data Availability

The original contributions presented in the study are publicly available. This data can be found here: https://doi.org/10.57760/sciencedb.17163.
